# Duration of health insurance coverage gaps and subsequent cost-related barriers to care among adults aged 18–64 years with multimorbidity

**DOI:** 10.3389/fpubh.2026.1965839

**Published:** 2026-09-10

**Authors:** Yongwen Cao, Zhendong Liu, Minmin Jiang, Tingting Ge, Lu Zhang, Yixi Liu, Xiuxia Li

**Affiliations:** 1 School of Public Health, Lanzhou University, Lanzhou, China; 2 Gansu University of Chinese Medicine, Lanzhou, China; 3 Gansu Provincial Hospital, Lanzhou, China; 4 Gansu Provincial Maternal and Child Health Hospital (Gansu Central Hospital), Lanzhou, China

**Keywords:** access to care, health insurance, multimorbidity, public health, uninsurance

## Abstract

**Objectives:**

To examine whether health insurance coverage-gap duration predicts subsequent cost-related barriers to care among working-age adults with multimorbidity.

**Methods:**

We pooled Medical Expenditure Panel Survey Panels 23 and 24, identifying 1,694 adults aged 18–64 years with at least two of nine chronic physical condition groups at baseline. Uninsured months during Years 1–2 were categorized as 0, 1–5, 6–23, or 24. The outcome was any cost-related delay in or inability to obtain medical care or prescription medicines during Years 3–4. Survey-weighted logistic regression accounted for weights, strata, primary sampling units, domain analysis, and Taylor-series linearization. Sensitivity analyses examined categorical age, insurance-source patterns, and cancer exclusion. Constant 2022 US-dollar expenditures were summarized using weighted medians and interquartile ranges and analyzed using two-part models with survey-aware jackknife confidence intervals for standardized means.

**Results:**

Among 1,694 participants, 379 experienced the outcome. Compared with no gap, adjusted odds ratios were 1.61 (95% CI, 0.94–2.77) for 1–5 uninsured months, 2.06 (95% CI, 1.17–3.63) for 6–23 months, and 3.44 (95% CI, 1.84–6.44) for 24 months; adjusted probabilities were 17.4, 24.8, 29.3, and 39.8%, respectively. Findings were robust to categorical age adjustment and cancer exclusion. Only continuous uninsurance was significantly associated with the outcome relative to continuous private coverage. For continuous uninsurance versus no gap, weighted median total expenditures were $1,235 versus $9,378, and median out-of-pocket expenditures were $464 versus $944. Adjusted total expenditures were lower ($4,884 vs. $26,134; ratio, 0.19; *p* < 0.001), whereas adjusted out-of-pocket expenditures did not differ significantly ($2,632 vs. $2,229; ratio, 1.18; *p* = 0.422).

**Conclusion:**

Longer coverage gaps were associated with greater subsequent cost-related barriers. Lower total spending without significantly lower out-of-pocket spending among continuously uninsured adults suggests foregone or reduced care rather than improved financial protection.

## 1 Introduction

Multimorbidity, commonly defined as the coexistence of two or more chronic conditions, is a growing challenge for clinical care and health policy in the United States (1, 2). It is not confined to older adults. In 2018, 27.2% of US adults had at least two of 10 selected chronic conditions, including 6.7% of adults aged 18–44 years and 33.0% of those aged 45–64 years (3–5). Adults with multiple chronic conditions often require recurring outpatient care, laboratory monitoring, and long-term medication treatment. Among adults who used health care in 2014, those with multiple chronic conditions incurred more than 3 times the total medical expenditures and more than twice the out-of-pocket expenditures of those without multiple chronic conditions (5). Consequently, affordable and continuous access to care is economically important throughout the full 18–64-year age range, well before Medicare eligibility.

Health insurance can reduce the effective price of care at the point of use and protect households from uncertain medical costs, but coverage among adults aged 18–64 years is often unstable. National Health Interview Survey data showed that adults with any period of noncoverage were substantially more likely to report an unmet medical need because of cost than adults with continuous coverage (6, 7). Longitudinal Medical Expenditure Panel Survey (MEPS) research likewise found that more than one-quarter of nonelderly adults experienced an insurance gap over a two-year period even after implementation of the Affordable Care Act, and that coverage gaps were associated with poorer access and lower use of preventive and other services (8). These constraints may be especially consequential for people with chronic disease. More than 40% of nonelderly adults with diabetes were in families reporting medical-bill hardship, with lack of insurance and greater comorbidity among the strongest correlates (9, 10). Cost-related medication nonadherence has also been associated with higher mortality among adults with diabetes, cardiovascular disease, or hypertension (11), and recent national estimates show that adults aged 18–64 years with four or more chronic conditions remain particularly vulnerable to such nonadherence (12).

Prior research has established that insurance instability changes when and where people obtain care, but several questions remain. Adults cycling out of and back into Medicaid deferred office-based visits while uninsured (13). In a separate MEPS cohort, people who had been uninsured for at least 1 year increased both office and emergency department use after obtaining full-year coverage, a pattern consistent with pent-up need (14). Other studies have generally classified coverage as current versus absent, continuous versus disrupted, or stable versus unstable (6, 8, 13, 14). Less is known about whether the cumulative duration of an early uninsured period is associated with affordability barriers measured later, particularly among adults already living with multiple physical chronic conditions. It is also unclear whether changing between private and public coverage without becoming uninsured carries the same risk as an actual gap in coverage.

A health-economics assessment of coverage gaps should distinguish financial protection from low medical spending. Uninsured adults use fewer recommended and therapeutic services than insured adults (15, 16), and experimental evidence shows that gaining coverage can increase health care use while reducing financial strain (17). Thus, lower expenditure after a coverage gap may reflect foregone or delayed care rather than improved economic protection. Self-reported delay or inability to obtain medical care or prescription medications because of cost captures a realized financial access constraint, whereas total and out-of-pocket expenditures describe different consequences of that constraint. Considering these outcomes together can help distinguish reduced consumption of care from reduced household financial exposure.

We therefore used two nationally representative four-year MEPS Household Component panels to examine adults aged 18–64 years with baseline physical multimorbidity. We assessed whether the number of uninsured months during Years 1–2 was associated with a cost-related barrier to medical care or prescription medications during Years 3–4. We also evaluated insurance-source patterns that separated uninterrupted changes in coverage source from true coverage gaps, tested the robustness of the findings to categorical age adjustment and exclusion of participants with cancer, and compared standardized total and out-of-pocket expenditures in constant 2022 US dollars.

## 2 Methods

### 2.1 Data source and study design

We conducted a pooled longitudinal analysis of Panels 23 and 24 of the Medical Expenditure Panel Survey Household Component (MEPS-HC). Panel 23 followed participants from 2018 through 2021, and Panel 24 followed participants from 2019 through 2022. We used the four-year longitudinal public-use files HC-236 and HC-245 and linked each record to the HC-036 pooled variance-estimation file by the unique combination of DUPERSID and PANEL. The linkage supplied the 1996–2023 combined variance stratum (STRA9623) and primary sampling unit (PSU9623) identifiers. All 12,645 longitudinal records matched, and no duplicate linkage keys were present. Because MEPS public-use data are publicly available and deidentified, institutional review board review and informed consent were not required for this secondary analysis.

### 2.2 Study population

Participants were required to be aged 18–61 years in Year 1 and no older than 64 years in Year 4 so that they remained within the 18-64-year age range throughout follow-up. We then required complete Year 1 ascertainment of all nine chronic physical condition groups and defined baseline multimorbidity as at least two groups. Eligible participants also needed complete monthly insurance information for all 24 months of Years 1–2, complete ascertainment of the cost-related barrier items in both the early (Years 1–2) and late (Years 3–4) windows, and nonmissing values for all covariates in the fully adjusted model. The final complete-case analytic sample included 1,694 participants. Sample disposition is shown in Table 1.

**Table 1:** Analytic sample flow.

Stage	Panel 23	Panel 24	Total	Excluded
Longitudinal records	7,080	5,565	12,645	—
Age eligible across 4 years	3,502	2,764	6,266	6,379
Complete ascertainment of 9 condition groups	3,500	2,763	6,263	3
Baseline physical multimorbidity (> = 2 groups)	975	761	1,736	4,527
Complete 24-month insurance exposure	972	760	1,732	4
Complete early and late outcome windows	954	749	1,703	29
Complete fully adjusted model	945	749	1,694	9

Excluded indicates the number removed from the immediately preceding stage.

### 2.3 Baseline physical multimorbidity

The 9 Year 1 condition groups were hypertension (HIBPDXY1), high cholesterol (CHOLDXY1), diabetes (DIABDXY1_M18), heart disease, stroke (STRKDXY1), emphysema (EMPHDXY1), cancer (CANCERY1), arthritis (ARTHDXY1), and asthma (ASTHDXY1). Heart disease was a composite defined as any affirmative response for coronary heart disease (CHDDXY1), angina (ANGIDXY1), myocardial infarction (MIDXY1), or other heart disease (OHRTDXY1); all four component responses were required. For every condition, a value of 1 was coded as present, 2 as absent, and negative MEPS response codes as missing. Complete ascertainment of all nine groups was required before the number of conditions was summed. The threshold of two or more conditions followed the standard multiple chronic conditions definition. These conditions were selected because they are common, policy-relevant chronic physical conditions with harmonized Year 1 indicators in both four-year longitudinal files.

Cancer was treated as one condition because the longitudinal files provide a harmonized Year 1 indicator for any cancer diagnosis, whereas cancer type, stage, treatment status, and duration are not represented consistently enough to support uniform stratification across both panels. We therefore evaluated this decision in a prespecified sensitivity analysis that excluded all participants with cancer.

### 2.4 Insurance coverage exposure

The primary exposure was the cumulative number of uninsured months during Years 1–2, derived from the 24 edited monthly insurance indicators INS [month] Y1X and INS [month] Y2X. A response of 1 denoted insured and 2 denoted uninsured. All 24 monthly responses were required. The count represented total uninsured months and did not require the months to be consecutive. We categorized the count as 0 months, 1–5 months, 6–23 months, or 24 months; the 0-month group was the reference. The 24-month category therefore represented continuous uninsurance throughout the exposure window.

### 2.5 Primary outcome

The primary outcome was any subsequent cost-related barrier to medical care or prescription medicines during Years 3–4. Four MEPS items asked whether the respondent delayed medical care because of cost (DLAYCA), could not afford medical care (AFRDCA), delayed obtaining prescription medicines because of cost (DLAYPM), or could not afford prescription medicines (AFRDPM). The items were administered in rounds 6 and 8 for the late outcome window, yielding eight item-round responses (DLAYCA6/8, AFRDCA6/8, DLAYPM6/8, and AFRDPM6/8). The binary outcome equaled 1 if any response was yes and 0 only if all eight responses were no; all eight responses were required. The analogous round 2 and 4 variables defined the early Years 1–2 ascertainment window, for which complete responses were required to maintain a uniformly observed four-year cohort; the primary outcome itself was based only on rounds 6 and 8.

### 2.6 Covariates and timing

The fully adjusted model included panel; baseline age in years (AGEY1X); sex (SEX); race and ethnicity (RACETHX: Hispanic, non-Hispanic White, non-Hispanic Black, non-Hispanic Asian, or non-Hispanic other/multiple races); Year 1 Federal Poverty Level (FPL) category (POVCATY1: poor/negative, near poor, low income, middle income, or high income); education when first entering MEPS (EDUCYR: less than high school, high school, some college, or bachelor’s degree or higher); Round 1 employment (EMPST1H: employed or had a job to return to vs. not employed); Round 1 marital status (MARRY1X: married vs. not married); Round 1 Census region (REGION1); Round 1 self-rated physical health (RTHLTH1: fair/poor vs. excellent/very good/good); Round 1 self-rated mental health (MNHLTH1, categorized identically); and the Year 1 nine-condition count. Sex and race and ethnicity were the MEPS edited/imputed characteristics, and education was the value recorded when the participant first entered MEPS. Later-round updates were not substituted for these baseline covariates.

### 2.7 Missing data

Variable-level missingness was evaluated among the 1,736 age-eligible participants with baseline multimorbidity. Missingness was low for every variable (0–1.27%). We used complete-case analysis for the variables required by each model and did not impute missing values. The complete-case sample for the fully adjusted primary model was 1,694. Variable-specific counts are reported in Supplementary Table.

### 2.8 Statistical analysis

We pooled the two longitudinal panels and divided the four-year longitudinal weight (LONGWT) by 2. Before restricting to the analytic cohort, we created the complex-survey design on all 12,645 pooled records using STRA9623, PSU9623, the pooled weight, and nesting of PSUs within strata. Analytic cohorts were then specified as survey domains. This design contained 117 strata and 294 PSU-stratum units, giving 177 denominator degrees of freedom. Taylor-series linearization was used for variances in the primary and sensitivity models. Singleton PSUs were handled with the survey package’s centered adjustment. All tests were 2-sided, and *p* < 0.05 was considered statistically significant.

We summarized baseline characteristics in the fully adjusted analytic sample overall and by coverage-gap category using unweighted sample sizes and survey-weighted means or percentages. We first estimated unadjusted survey-weighted outcome prevalence by coverage-gap category. The primary association was estimated with a survey-weighted multivariable logistic regression with a quasibinomial variance function and the covariates specified above. Results are reported as adjusted odds ratios (ORs) with 95% confidence intervals (CIs). Covariate-standardized probabilities were obtained by setting every participant’s exposure to each gap category in turn, predicting the outcome, and averaging the predictions with the survey weights. Their CIs used the model covariance matrix and the delta method. Wald CIs and *p* values used the full pooled-design 177 degrees of freedom.

#### 2.8.1 Sensitivity analyses

First, we replaced continuous age with categories of 18–34, 35–49, and 50–64 years. Second, we separated zero-gap participants by insurance source using the 24 monthly private (PRI [month]Y1/Y2) and public (PUB [month]Y1X/Y2X) indicators. The reference group had private coverage in all 24 months; this category could include concurrent public coverage. The public-only group had no private coverage and public coverage in all 24 months. Other fully insured participants whose source pattern changed were classified as source switch without a gap. Participants with uninsured months retained the 1–5, 6–23, and 24-month categories. Public coverage included Medicare, Medicaid, TRICARE, and other public programs represented by the MEPS public-insurance indicators. Third, we repeated the primary model after excluding participants with any Year 1 cancer diagnosis.

#### 2.8.2 Supplementary health economics analysis

We examined cumulative total health-care expenditures (TOTEXPY3 plus TOTEXPY4) and out-of-pocket expenditures paid by the respondent or family (TOTSLFY3 plus TOTSLFY4) during Years 3–4. Panel 23 Years 3 and 4 corresponded to 2020 and 2021, whereas Panel 24 Years 3 and 4 corresponded to 2021 and 2022. Before summing the two annual amounts, we converted each to constant 2022 United States dollars following Agency for Healthcare Research and Quality guidance for pooled MEPS expenditures. Total expenditures were adjusted with the Personal Consumption Expenditures Health (PCE-Health) index, and out-of-pocket expenditures with the medical care Consumer Price Index (CPI-M), by multiplying each annual value by the 2022 index divided by the index for its calendar year.

Because expenditure distributions included zeros and were right-skewed, we fitted a two-part model for each outcome: a survey-weighted logistic model for any expenditure and, among participants with positive expenditures, a survey-weighted generalized linear model with a gamma distribution and log link. Both components used the primary exposure and adjustment set. For each gap category, the standardized unconditional mean was the survey-weighted average of the product of the predicted probability of any expenditure and predicted positive expenditure. Joint uncertainty was estimated from a stratified delete-one-PSU jackknife (JKn) replicate design generated from the pooled strata and PSU structure. Both parts were refitted and the standardized means recalculated in each of 294 replicates. We report joint 95% CIs and direct mean differences and mean ratios relative to the no-gap group; ratio-based CIs and tests were computed on the log scale using 177 degrees of freedom. To describe the skewed distributions, we additionally calculated survey-weighted unconditional medians and interquartile ranges within each coverage-gap category, including participants with zero expenditures. These medians and interquartile ranges were descriptive estimates and were not model adjusted; statistical inference was based on the two component models and the adjusted standardized unconditional means.

All final analyses were performed in R version 4.5.0 using the haven version 2.5.5 and survey version 4.5 packages.

## 3 Results

### 3.1 Study population and missingness

The pooled longitudinal files contained 12,645 records (7,080 in Panel 23 and 5,565 in Panel 24). Of these, 6,266 met the age criteria, 6,263 had complete ascertainment of all nine condition groups, and 1,736 had baseline physical multimorbidity. After requiring complete monthly insurance exposure (*n* = 1,732), complete early and late outcome windows (*n* = 1,703), and all model covariates, 1,694 participants remained in the fully adjusted analysis (Table 1).

Among the 1,736 age-eligible participants with multimorbidity, no data were missing for age, sex, race and ethnicity, FPL category, condition count, expenditures, or survey design variables. Missingness was 0.52% for education, 0.17% each for employment, marital status, region, and self-rated physical health, 0.29% for self-rated mental health, 0.23% for the 24-month insurance exposure, 0.46% for the early barrier window, and 1.27% for the late outcome.

In the outcome-complete cohort of 1,703 participants, 1,346 had no uninsured months, 130 had 1–5 uninsured months, 150 had 6–23 uninsured months, and 77 were uninsured for all 24 months. The weighted shares were 82.7, 6.7, 7.5, and 3.2%, respectively. The continuously uninsured group included 40 participants with the outcome, and estimates for this small group were correspondingly less precise. Baseline characteristics of the fully adjusted analytic sample overall and by coverage-gap category are shown in Table 2. In that sample, 11.0% were aged 18–34 years, 29.8% were aged 35–49 years, and 59.2% were aged 50–64 years on a survey-weighted basis.

**Table 2:** Baseline characteristics of the fully adjusted analytic sample overall and by coverage-gap duration.

Characteristic	Overall (*N* = 1,694)	0 months (*n* = 1,340)	1–5 months (*n* = 128)	6–23 months (*n* = 149)	24 months (*n* = 77)
Age group, years, 18–34	146 (11.0)	100 (9.8)	22 (18.1)	19 (21.0)	5 (3.6)
35–49	501 (29.8)	378 (28.7)	40 (32.8)	54 (32.2)	29 (44.6)
50–64	1,047 (59.2)	862 (61.5)	66 (49.1)	76 (46.7)	43 (51.8)
Sex, Male	696 (47.1)	565 (47.2)	45 (46.9)	56 (44.1)	30 (49.9)
Female	998 (52.9)	775 (52.8)	83 (53.1)	93 (55.9)	47 (50.1)
Race and ethnicity, Hispanic	329 (13.8)	201 (11.1)	40 (27.4)	47 (20.8)	41 (40.6)
Non-Hispanic White	885 (65.0)	760 (68.1)	55 (52.7)	50 (51.1)	20 (41.9)
Non-Hispanic Black	358 (13.8)	280 (13.2)	27 (15.7)	38 (19.2)	13 (15.0)
Non-Hispanic Asian	45 (3.2)	34 (3.2)	3 (3.1)	8 (4.9)	0 (0.0)
Non-Hispanic other or multiple races	77 (4.1)	65 (4.4)	3 (1.0)	6 (4.0)	3 (2.4)
Federal Poverty Level category, Poor or negative income	389 (14.9)	291 (13.0)	29 (17.1)	39 (26.0)	30 (33.8)
Near poor	90 (3.8)	65 (3.6)	9 (6.4)	12 (5.4)	4 (2.2)
Low income	239 (12.6)	169 (11.3)	19 (16.8)	33 (21.1)	18 (17.7)
Middle income	455 (25.7)	356 (25.0)	34 (22.7)	48 (33.3)	17 (32.9)
High income	521 (43.0)	459 (47.2)	37 (37.0)	17 (14.2)	8 (13.5)
Educational attainment, Less than high school	329 (12.2)	226 (10.3)	20 (6.9)	52 (30.0)	31 (32.8)
High school	541 (28.2)	421 (27.8)	43 (30.4)	51 (29.9)	26 (29.0)
Some college	406 (27.4)	336 (28.1)	31 (27.3)	26 (21.5)	13 (23.0)
Bachelor’s degree or higher	418 (32.2)	357 (33.8)	34 (35.5)	20 (18.7)	7 (15.3)
Employed, yes	1,052 (71.8)	817 (72.2)	96 (78.3)	93 (64.4)	46 (65.1)
Married, yes	767 (55.0)	631 (57.7)	45 (40.0)	61 (40.4)	30 (50.0)
Census region, Northeast	282 (17.5)	241 (19.0)	18 (8.6)	20 (13.9)	3 (6.2)
Midwest	332 (22.2)	275 (23.0)	20 (17.9)	25 (20.4)	12 (13.7)
South	703 (39.7)	524 (37.9)	55 (43.4)	74 (46.0)	50 (63.0)
West	377 (20.6)	300 (20.1)	35 (30.2)	30 (19.7)	12 (17.1)
Fair or poor physical health, yes	563 (26.7)	436 (24.9)	36 (28.2)	59 (38.4)	32 (43.6)
Fair or poor mental health, yes	281 (13.7)	221 (12.9)	19 (13.5)	26 (20.0)	15 (18.9)
Number of baseline physical condition groups, mean	2.8	2.8	2.8	2.7	2.5
Cost-related barrier during Years 1–2, yes	528 (27.9)	333 (22.7)	62 (41.0)	87 (61.0)	46 (56.5)
MEPS panel, Panel 23	945 (52.8)	737 (52.5)	78 (56.3)	83 (52.6)	47 (51.8)
Panel 24	749 (47.2)	603 (47.5)	50 (43.7)	66 (47.4)	30 (48.2)

Values are unweighted frequencies, *n* (survey-weighted percentages), unless otherwise indicated. Sample sizes in the column headings are unweighted. The number of baseline physical condition groups is presented as a survey-weighted mean. Percentages may not sum to 100.0 because of rounding. FPL indicates Federal Poverty Level; MEPS, Medical Expenditure Panel Survey.

### 3.2 Coverage-gap duration and subsequent cost-related barriers

Unadjusted survey-weighted prevalence of a cost-related barrier during Years 3–4 rose from 16.9% (95% CI, 14.2–19.7%) among participants with no uninsured months to 27.3% (95% CI, 16.9–37.8%) for 1–5 months, 33.4% (95% CI, 21.3–45.5%) for 6–23 months, and 43.9% (95% CI, 29.1–58.8%) for 24 months (Table 3).

**Table 3:** Coverage-gap duration and subsequent cost-related barriers.

Gap duration	*n*	Events	Weighted % (95% CI)	Adjusted OR (95% CI)	Adjusted % (95% CI)	*p* value
0 months	1,346	246	16.9 (14.2–19.7)	Reference	17.4 (14.9–20.3)	-
1–5 months	130	39	27.3 (16.9–37.8)	1.61 (0.94–2.77)	24.8 (17.2–34.3)	0.083
6–23 months	150	56	33.4 (21.3–45.5)	2.06 (1.17–3.63)	29.3 (20.3–40.2)	0.012
24 months	77	40	43.9 (29.1–58.8)	3.44 (1.84–6.44)	39.8 (27.9–53.0)	<0.001

Unadjusted *n*, events, and weighted prevalence use the outcome-complete cohort (*N* = 1,703). Adjusted ORs and standardized probabilities use the complete-case model (*N* = 1,694; 379 events) and control for panel, age, sex, race and ethnicity, Year 1 FPL category, education, employment, marital status, region, self-rated physical and mental health, and condition count. CI indicates confidence interval; OR, odds ratio.

After multivariable adjustment, compared with no uninsured months, the OR was 1.61 (95% CI, 0.94–2.77; *p* = 0.083) for 1–5 months, 2.06 (95% CI, 1.17–3.63; *p* = 0.012) for 6–23 months, and 3.44 (95% CI, 1.84–6.44; *p* < 0.001) for 24 months. The corresponding standardized probabilities were 17.4% (95% CI, 14.9–20.3%), 24.8% (95% CI, 17.2–34.3%), 29.3% (95% CI, 20.3–40.2%), and 39.8% (95% CI, 27.9–53.0%), respectively. Thus, the adjusted absolute probability was approximately 22 percentage points higher for continuous uninsurance than for no gap.

### 3.3 Sensitivity analyses

Replacing continuous age with the three age categories produced nearly identical estimates: ORs were 1.62 (95% CI, 0.94–2.78), 2.08 (95% CI, 1.19–3.63), and 3.45 (95% CI, 1.83–6.48) for 1–5, 6–23, and 24 uninsured months, respectively (Table 4).

**Table 4:** Sensitivity analyses for the cost-related barrier outcome.

Analysis	Contrast	Adjusted OR	95% CI	*p*-value	*N* (events)
Categorical age	1–5 vs. 0 months	1.62	0.94–2.78	0.081	1,694 (379)
Categorical age	6–23 vs. 0 months	2.08	1.19–3.63	0.010	1,694 (379)
Categorical age	24 vs. 0 months	3.45	1.83–6.48	<0.001	1,694 (379)
Insurance source	Public only, no gap vs. private throughout	0.56	0.33–0.96	0.036	1,694 (379)
Insurance source	Source switch, no gap vs. private throughout	0.68	0.28–1.65	0.390	1,694 (379)
Insurance source	1-5-month gap vs. private throughout	1.32	0.74–2.35	0.346	1,694 (379)
Insurance source	6-23-month gap vs. private throughout	1.61	0.86–3.02	0.139	1,694 (379)
Insurance source	24-month gap vs. private throughout	2.71	1.38–5.33	0.004	1,694 (379)
Cancer exclusion	1–5 vs. 0 months	1.99	1.06–3.74	0.032	1,441 (312)
Cancer exclusion	6–23 vs. 0 months	2.21	1.25–3.89	0.006	1,441 (312)
Cancer exclusion	24 vs. 0 months	3.72	1.83–7.55	<0.001	1,441 (312)

All models used the same adjustment set as the primary analysis, except that the categorical-age model replaced continuous age with 18–34, 35–49, and 50–64 years.

In the insurance-source analysis, compared with private coverage throughout and no gap, the OR was 0.56 (95% CI, 0.33–0.96; *p* = 0.036) for public-only coverage throughout and 0.68 (95% CI, 0.28–1.65; *p* = 0.390) for a source switch without an uninsured month. The ORs for 1–5, 6–23, and 24 uninsured months were 1.32 (95% CI, 0.74–2.35), 1.61 (95% CI, 0.86–3.02), and 2.71 (95% CI, 1.38–5.33), respectively; only the 24-month estimate was statistically significant.

After exclusion of participants with cancer, 1,441 participants with 312 outcome events remained. The ORs were 1.99 (95% CI, 1.06–3.74; *p* = 0.032), 2.21 (95% CI, 1.25–3.89; *p* = 0.006), and 3.72 (95% CI, 1.83–7.55; *p* < 0.001) for 1–5, 6–23, and 24 uninsured months, respectively. The graded association therefore persisted after removing the heterogeneous cancer group.

### 3.4 Supplementary expenditure outcomes

Survey-weighted median (IQR) total health-care expenditures across Years 3–4 were $9,378 ($3,007–$30,391) in the no-gap group, $5,923 ($2,869–$20,681) for 1–5 uninsured months, $4,623 ($920–$17,791) for 6–23 months, and $1,235 ($91–$6,487) for 24 months. In standardized two-part models expressed in constant 2022 United States dollars, the corresponding adjusted means were $26,134 (95% CI, $23,092–$29,575), $20,056 (95% CI, $13,948–$28,839), $20,935 (95% CI, $14,508–$30,209), and $4,884 (95% CI, $3,286–$7,260), respectively (Table 5). Compared with no gap, the 24-month group had a mean ratio of 0.19 (95% CI, 0.12–0.28; *p* < 0.001) and a mean difference of -$21,249 (95% CI, −$24,927 to -$17,571; *p* < 0.001). Direct contrasts for the 1–5- and 6-23-month groups were not statistically significant.

**Table 5:** Years 3–4 expenditures by coverage-gap duration.

Outcome	Gap	Weighted median (IQR)	Adjusted mean (95% CI)	Mean difference (95% CI)	Mean ratio (95% CI)	*p*-value
Total	0 months	$9,378 ($3,007–$30,391)	$26,134 ($23,092–$29,575)	Reference	Reference	—
Total	1–5 months	$5,923 ($2,869–$20,681)	$20,056 ($13,948–$28,839)	-$6,078 (−$13,611 to $1,456)	0.77 (0.53–1.12)	0.168
Total	6–23 months	$4,623 ($920–$17,791)	$20,935 ($14,508–$30,209)	-$5,199 (−$13,297 to $2,899)	0.80 (0.55–1.17)	0.254
Total	24 months	$1,235 ($91–$6,487)	$4,884 ($3,286–$7,260)	-$21,249 (−$24,927 to -$17,571)	0.19 (0.12–0.28)	<0.001
Out of pocket	0 months	$944 ($237–$2,598)	$2,229 ($1,982–$2,507)	Reference	Reference	-
Out of pocket	1–5 months	$703 ($124–$2,175)	$1,902 ($1,424–$2,542)	-$327 (−$945 to $291)	0.85 (0.62–1.17)	0.327
Out of pocket	6–23 months	$479 ($103–$1,656)	$2,812 ($2,095–$3,774)	$582 (−$278 to $1,442)	1.26 (0.92–1.73)	0.146
Out of pocket	24 months	$464 ($4–$1,871)	$2,632 ($1,769–$3,917)	$403 (−$661 to $1,467)	1.18 (0.79–1.77)	0.422

Amounts are cumulative across Years 3–4 and expressed in constant 2022 United States dollars. Survey-weighted medians and interquartile ranges are unadjusted descriptive estimates that include zero expenditures. Adjusted means, joint 95% CIs, and direct contrasts were estimated from the two-part models using 294 stratified JKn replicates. Total expenditures were adjusted with PCE-Health and out-of-pocket expenditures with CPI-M before annual values were summed. *p*-values shown are for adjusted mean ratios versus 0 months. CI indicates confidence interval; IQR, interquartile range.

The two component models showed that continuous uninsurance was associated with both a lower probability of any total expenditure (OR, 0.21; 95% CI, 0.07–0.60; *p* = 0.004) and lower expenditure among participants with positive spending (conditional mean ratio, 0.20; 95% CI, 0.13–0.29; *p* < 0.001) (Table 6). The 6-23-month group also had a lower probability of any total expenditure (OR, 0.31; 95% CI, 0.10–0.97; *p* = 0.044), although its standardized unconditional mean did not differ significantly from the no-gap group.

**Table 6:** Components of the two-part expenditure models.

Outcome	Model component	Gap vs 0 months	Effect	95% CI	*p* value
Total	Any spending (OR)	1–5 months	1.58	0.44–5.74	0.484
Total	Any spending (OR)	6–23 months	0.31	0.10–0.97	0.044
Total	Any spending (OR)	24 months	0.21	0.07–0.60	0.004
Total	Positive spending (CMR)	1–5 months	0.76	0.54–1.08	0.129
Total	Positive spending (CMR)	6–23 months	0.83	0.58–1.18	0.290
Total	Positive spending (CMR)	24 months	0.20	0.13–0.29	<0.001
Out of pocket	Any spending (OR)	1–5 months	0.70	0.34–1.41	0.313
Out of pocket	Any spending (OR)	6–23 months	0.69	0.28–1.72	0.427
Out of pocket	Any spending (OR)	24 months	0.30	0.10–0.89	0.030
Out of pocket	Positive spending (CMR)	1–5 months	0.87	0.64–1.19	0.374
Out of pocket	Positive spending (CMR)	6–23 months	1.28	0.97–1.70	0.082
Out of pocket	Positive spending (CMR)	24 months	1.29	0.91–1.81	0.146

OR indicates odds ratio; CMR, conditional mean ratio among participants with positive expenditures. All component-model variances used Taylor-series linearization.

Survey-weighted median (IQR) out-of-pocket expenditures were $944 ($237–$2,598) in the no-gap group, $703 ($124–$2,175) for 1–5 uninsured months, $479 ($103–$1,656) for 6–23 months, and $464 ($4–$1,871) for 24 months. The corresponding adjusted standardized means were $2,229 (95% CI, $1,982–$2,507), $1,902 (95% CI, $1,424–$2,542), $2,812 (95% CI, $2,095–$3,774), and $2,632 (95% CI, $1,769–$3,917), respectively. None of the direct out-of-pocket mean ratios or differences was statistically significant. Continuous uninsurance was associated with a lower probability of any out-of-pocket spending (OR, 0.30; 95% CI, 0.10–0.89; *p* = 0.030), but the positive-spending conditional mean ratio was not significant (1.29; 95% CI, 0.91–1.81; *p* = 0.146). The combination of markedly lower total spending and no corresponding reduction in adjusted out-of-pocket spending is consistent with reduced use or foregone care rather than improved financial protection.

## 4 Discussion

In this longitudinal, nationally representative analysis of adults aged 18–64 years with baseline physical multimorbidity, longer uninsured periods during Years 1–2 were associated with higher probabilities of a cost-related barrier during Years 3–4. Compared with no uninsured months, adjusted odds ratios were 1.61 for a 1–5-month gap, 2.06 for a 6–23-month gap, and 3.44 for 24 months without coverage. The estimate for the shortest gap did not reach conventional statistical significance, but the point estimates and standardized probabilities showed a graded pattern across categories. The adjusted probability increased from 17.4% with no gap to 39.8% with 24 uninsured months, an absolute difference of 22.4 percentage points. This most prolonged gap was also associated with markedly lower subsequent total medical spending, while adjusted out-of-pocket spending was not significantly lower.

These findings extend prior evidence on coverage continuity. Earlier national studies linked part-year or unstable coverage to cost-related unmet need, reduced preventive care, and changes in office and emergency department use (13, 14). The present analysis adds four features. It focuses on adults with established need for ongoing care; measures the exposure month by month over 2 years; preserves temporal ordering by assessing barriers in the subsequent 2 years; and distinguishes uninterrupted switching between private and public sources from periods with no coverage. The results therefore suggest that duration contains information that is lost when instability is represented only as a binary indicator. At the same time, because this was an observational study and the shortest-gap estimate was imprecise, the pattern should not be interpreted as proof of a causal duration gradient.

The expenditure results are central to the economic interpretation. For participants uninsured throughout Years 1–2, the standardized mean total expenditure in Years 3–4 was $4,884, compared with $26,134 among those with no gap (mean ratio, 0.19; adjusted difference, −$21,249). The two-part model indicated both a lower probability of any total expenditure and substantially lower spending among people with positive expenditures. In contrast, the standardized out-of-pocket means were $2,632 and $2,229, respectively, with no statistically significant difference. Among participants with positive out-of-pocket spending, the conditional mean ratio was 1.29, although its confidence interval included the null. Read together with the higher prevalence of reported cost barriers, this pattern is compatible with suppressed use or foregone care rather than improved financial protection. It is also consistent with prior evidence that uninsured adults receive fewer services, whereas gaining coverage can increase utilization and reduce financial strain (5, 15–17). The expenditure models do not identify which services were forgone, so this explanation remains an interpretation rather than a directly tested mechanism.

The insurance-source sensitivity analysis further separates continuity from source. Uninterrupted switching between private and public coverage was not associated with higher odds of a later cost-related barrier relative to private coverage throughout. Public-only coverage without a gap was associated with lower odds, while the 24-month uninsured category remained associated with higher odds. Other studies have found meaningful differences in access and financial strain across Medicaid, Marketplace, and employer-sponsored plans, and randomized evidence indicates that Medicaid can reduce financial strain (18). However, our public category combines heterogeneous programs, the private-throughout reference may include months of concurrent public coverage, and plan generosity and eligibility are not randomly assigned. The source-specific estimates should therefore be viewed as supportive sensitivity results, not as causal comparisons of public and private insurance or as Medicaid-specific effects.

Robustness analyses support the main inference while defining its boundaries. Replacing continuous age with categories of 18–34, 35–49, and 50–64 years produced nearly identical estimates, indicating that the findings were not driven by an assumed linear age effect. After participants with cancer were excluded, all three gap categories were associated with higher odds of the outcome, and the estimate for 24 uninsured months remained large. This reduces concern that broad cancer ascertainment or cancer-related treatment costs alone explain the primary association. Nevertheless, the continuously uninsured group contained only 77 participants and 40 outcome events in the primary cohort, so its confidence intervals remain appropriately wide.

Several mechanisms may account for persistence of affordability barriers after the exposure period. During an uninsured spell, adults may postpone visits, monitoring, or prescription fills, interrupt relationships with clinicians, or lose access to negotiated prices and care coordination. Prior MEPS studies document deferred office care during coverage loss and increased use after long periods of uninsurance (19). For people managing several chronic conditions, accumulated unmet need may increase the complexity and price of later care. Medical bills incurred without coverage may also compete with future health and nonhealth spending. The strong associations of medical-bill hardship and cost-related medication nonadherence with adverse outcomes make these pathways clinically and economically relevant (9, 11). Our data support the presence of later financial access constraints but cannot distinguish among these mechanisms.

The policy implication is broader than any single insurance program: continuity itself is an important dimension of coverage quality. Income changes, employment transitions, eligibility rules, renewal procedures, and movement between public and private markets can all create gaps. Modeling studies have shown that eligibility changes between Medicaid and Marketplace coverage are common and can be mitigated by policies that improve continuity across programs (19–21). Empirical evaluations further indicate that Affordable Care Act Medicaid expansions reduced coverage disruptions and improved coverage, access, affordability, and chronic-disease care among low-income adults (22–24). The current findings support efforts to prevent avoidable termination, simplify renewal, coordinate transitions between coverage sources, and provide timely enrollment assistance. Because the primary exposure combined gaps arising from any insurance source, these implications apply to the broader coverage system rather than to Medicaid alone.

This study has several strengths. The 4-year panel structure established a two-year exposure window before a separate 2-year outcome window. Month-level insurance information supported clinically interpretable duration categories, and the analysis accounted for MEPS weights, strata, primary sampling units, pooled-panel weights, and domain estimation. Standardized probabilities translated odds ratios into absolute risks. The supplementary two-part expenditure models used constant 2022 dollars and survey-aware jackknife confidence intervals for unconditional means and contrasts. Results were also examined under alternative age specification, insurance-source classification, and cancer exclusion.

Several limitations warrant emphasis. First, the study is observational. Residual confounding by job loss, income volatility, changes in health status, or other shocks may contribute to both uninsured months and later barriers; the estimates should not be interpreted as causal. Second, insurance and barrier measures were self-reported, and the outcome combines medical- and prescription-care items. Third, multimorbidity was defined using nine physical condition groups; mental health conditions were not counted, disease severity was unavailable, and the cancer indicator was broad. Fourth, complete-case analysis was used, although variable-level missingness was low. Fifth, the 6-23-month category encompassed a broad range of exposure durations and included only 150 participants and 56 outcome events in the outcome-complete cohort. Estimates for this category should therefore be interpreted cautiously, and the limited sample size precluded a more granular assessment of heterogeneity or duration-response patterns among longer gaps. The 24-month group was also small, with 77 participants and 40 outcome events. Sixth, insurance-source categories were necessarily heterogeneous and do not capture benefit design, deductibles, or provider networks. Seventh, the expenditure analysis did not decompose changes by service type or measure unmet clinical need directly. Eighth, coverage during Years 3–4 was not included as a covariate because it could mediate the association between an earlier gap and later barriers; accordingly, the results describe the prognostic significance of prior coverage history, not an effect independent of subsequent coverage. Finally, observation years overlapped the COVID-19 pandemic and related policy changes. Adjustment for MEPS panel addresses average panel differences but cannot fully separate pandemic-era changes in care seeking, coverage, and affordability.

## 5 Conclusion

Among US adults aged 18–64 years with multiple physical chronic conditions, longer uninsured periods were associated with substantially greater subsequent cost-related barriers to medical or prescription care. Continuous uninsurance during the two-year exposure period was associated with an adjusted barrier probability of 39.8%, compared with 17.4% among adults with no gap. The same group had much lower subsequent total medical spending without a corresponding reduction in adjusted out-of-pocket spending, a pattern compatible with constrained use rather than better financial protection.

## Data Availability

Publicly available datasets were analyzed in this study. This data can be found at: https://meps.ahrq.gov/mepsweb/.
