# Supplementary material for: Duration of health insurance coverage gaps and subsequent cost-related barriers to care among adults aged 18–64 years with multimorbidity
Source: Front Public Health. 2026 Sep 10;14:1965839. doi: 10.3389/fpubh.2026.1965839 (PMC13600860; doi:10.3389/fpubh.2026.1965839)
Supplement: Supplementary file 1 [file Table_1.docx]

Supplementary Table. Variable-level missingness among age-eligible adults with baseline multimorbidity (N=1,736)

| **Variable** | **Missing, n (%)** | **Handling** |
| --- | --- | --- |
| Age at baseline | 0 (0.00%) | No missing values |
| Sex | 0 (0.00%) | No missing values |
| Race and ethnicity | 0 (0.00%) | No missing values |
| Federal Poverty Level category | 0 (0.00%) | No missing values |
| Education | 9 (0.52%) | Complete case |
| Employment status | 3 (0.17%) | Complete case |
| Marital status | 3 (0.17%) | Complete case |
| Census region | 3 (0.17%) | Complete case |
| Self-rated physical health | 3 (0.17%) | Complete case |
| Self-rated mental health | 5 (0.29%) | Complete case |
| Nine-condition count | 0 (0.00%) | No missing values |
| 24-month insurance exposure | 4 (0.23%) | Complete case |
| Years 1-2 early barrier window | 8 (0.46%) | Complete case |
| Years 3-4 primary outcome | 22 (1.27%) | Complete case |
| Years 3-4 total expenditure | 0 (0.00%) | No missing values |
| Years 3-4 out-of-pocket expenditure | 0 (0.00%) | No missing values |
| Survey weight, stratum, and PSU | 0 (0.00%) | No missing values |

*Note: PSU indicates primary sampling unit. Missing values were not imputed.*
